# Vigilância em saúde pós-desastre da barragem da Samarco: avaliação dos padrões de assistência em doenças crônicas e transtornos mentais (2008-2023)

**DOI:** 10.1590/0102-311XPT193725

**Published:** 2026-08-10

**Authors:** Gederson Santos Rigoni, Andrea Sobral, Jonas Eduardo Monteiro dos Santos, Andre Reynaldo Santos Périssé

**Affiliations:** 1 Escola Nacional de Saúde Pública Sergio Arouca, Fundação Oswaldo Cruz, Fiocruz, Rio de Janeiro, Brasil.

**Keywords:** Desastres Ambientais, Doenças Crônicas, Transtornos Mentais, Barragens, Environmental Disasters, Chronic Diseases, Mental Disorders, Dams, Desastres Ambientales, Enfermedades Crónicas, Trastornos Mentales, Represas

## Abstract

O presente estudo comparou os possíveis impactos causados pelo rompimento da barragem da Samarco (Minas Gerais, Brasil) e municípios com perfil semelhante, com o objetivo analisar os padrões de morbimortalidade por doenças crônicas não transmissíveis em municípios atingidos. Trata-se de um estudo ecológico baseado em dados secundários de acesso público (SIA/SUS, SIH/SUS e SIM) entre os anos de 2008 a 2023. As análises foram realizadas por capítulos e grupos de doenças (circulatório, diabetes, respiratório, neoplasias e transtornos mentais) e estrato populacional. Foram calculadas taxas por 100 mil habitantes, diferenças percentuais e risco relativo, todos com intervalos de 95% de confiança. O período “pós-rompimento” (2016-2019) foi comparado aos períodos “antes do rompimento” (2008-2015) e “pós-COVID-19” (2020-2023). Observou-se aumento nas taxas de atendimentos ambulatoriais nos municipios atingidos em relação aos municípios de comparação, com destaque para diabetes (estratos 1, 2, 3 e 5), doenças respiratórias (estratos 1, 3, 4 e 5), transtornos mentais (estratos 4 e 5) e neoplasias (estratos 1, 2, 3 e 4). Nas internações, observaram-se aumentos discretos por problemas respiratórios no estrato 4 e neoplasias nos estratos 3, 4 e 5. Já em mortalidade, houve elevação nas taxas por transtornos mentais nos estratos 2 e 4. Os achados sugerem mudanças nos padrões assitenciais nos municípios atingidos, com possível sobrecarga pós-desastre, e reforçam a importância da vigilância e integração entre políticas de saúde, ambientais e gestão de riscos.

## Abstract (en)

This study compared the possible impacts caused by the Samarco dam collapse (Minas Gerais State, Brazil) and municipalities with a similar profile to analyze the morbidity and mortality patterns due to chronic noncommunicable diseases in the affected municipalities. This ecological study was based on publicly available secondary data (SIA/SUS, SIH/SUS, and SIM) from 2008 to 2023. The analyses were carried out by disease chapters and groups (circulatory, diabetes, respiratory, neoplasms, and mental disorders) and population strata. Rates per 100,000 inhabitants, percentage differences, and relative risk were calculated with 95% confidence intervals. The “post-collapse” period (2016-2019) was compared to the “before the collapse” (2008-2015) and “post-COVID-19” (2020-2023) ones. An increase in the rates of outpatient care occurred in the affected municipalities in relation to the comparison municipalities, especially regarding diabetes (strata 1, 2, 3, and 5), respiratory diseases (strata 1, 3, 4, and 5), mental disorders (strata 4 and 5), and neoplasms (strata 1, 2, 3, and 4). In hospitalizations, slight increases due to respiratory problems occurred in stratum 4 and due to neoplasms in strata 3, 4, and 5. Regarding mortality, this study found an increase in the rates of mental disorders in strata 2 and 4. These findings suggest changes in care patterns in the affected municipalities with possible post-disaster overload and reinforce the importance of surveillance and integration between health, environmental, and risk management policies.

## Resumen

Este estudio comparó los posibles impactos causados por la rotura de la presa de Samarco (Minas Gerais, Brasil) en municipios con perfiles similares, con el objetivo de analizar los estándares de morbimortalidad por enfermedades crónicas no transmisibles en los municipios afectados. Se trata de un estudio ecológico basado en datos secundarios de acceso público (SIA/SUS, SIH/SUS y SIM) correspondientes al periodo comprendido entre el 2008 y el 2023. Los análisis se realizaron por capítulos y grupos de enfermedades (circulatorias, diabetes, respiratorias, neoplasias y trastornos mentales) y por estrato de población. Se calcularon las tasas por cada 100.000 habitantes, las diferencias percentuales y el riesgo relativo, todas con intervalos de 95% de confianza. El período “posterior a la ruptura” (2016-2019) fue comparado con los períodos “antes de la ruptura” (2008-2015) y “posterior a la COVID-19” (2020-2023). Se observó un aumento en las tasas de atenciones ambulatorias en los municipios afectados en relación con los municipios de comparación, particularmente para la diabetes (estratos 1, 2, 3 y 5), enfermedades respiratorias (estratos 1, 3, 4 y 5), trastornos mentales (estratos 4 y 5) y neoplasias (estratos 1, 2, 3 y 4). En las hospitalizaciones, se observaron ligeros aumentos de problemas respiratorios en el estrato 4 y neoplasias en los estratos 3, 4 y 5. En cuanto a la mortalidad, hubo un aumento en las tasas debido a trastornos mentales en los estratos 2 y 4. Los resultados sugieren cambios en los estándares asistenciales en los municipios afectados, con una posible sobrecarga posterior al desastre, y refuerzan la importancia de la vigilancia y la integración entre las políticas de salud, medio ambiente y gestión de riesgos.

## Introdução

O rompimento da barragem de Fundão, pertencente à mineradora Samarco (empreendimento conjunto da Vale e da anglo-australiana BHP Billiton), ocorrido em 5 de novembro de 2015 na cidade de Mariana, em Minas Gerais, é considerado o maior desastre socioambiental do Brasil e foi classificado pela Organização das Nações Unidas (ONU) como uma violação de direitos humanos ^1^.

O desastre resultou no lançamento de cerca de 45 milhões de metros cúbicos de rejeitos no meio ambiente, causou a morte de 19 pessoas, deixou mais de 600 desabrigadas e contaminou aproximadamente 663km de cursos d’água ao longo do Rio Doce, desencadeando uma tragédia com impactos profundos nos âmbitos socioeconômico, ambiental e de infraestrutura em cerca de 40 municípios da Bacia do Rio Doce, considerados como atingidos ^2^. Entre os diversos efeitos, destacam-se os danos à saúde, com comprometimentos significativos no acesso e na qualidade da assistência, afetando diretamente a oferta e o funcionamento de serviços essenciais ^1^ ^,^ ^2^ ^,^ ^3^ ^,^ ^4^ ^,^ ^5^ ^,^ ^6^ ^,^ ^7^.

Estudos epidemiológicos indicam que exposições ambientais decorrentes de desastres podem resultar em efeitos adversos à saúde no curto, médio e longo prazos, incluindo o aumento do risco de doenças crônicas não transmissíveis (DCNT) e transtornos mentais ^3^ ^,^ ^4^ ^,^ ^5^ ^,^ ^6^ ^,^ ^7^ ^,^ ^8^ ^,^ ^9^. Tais efeitos decorrem não apenas da exposição a contaminantes e da desorganização dos serviços de saúde, mas também das mudanças nas condições de vida, alimentação, habitação e trabalho impostas às populações atingidas. Essas condições podem se manifestar progressivamente ao longo do tempo, exigindo estratégias de vigilância que considerem os determinantes sociais da saúde, os impactos ambientais persistentes e as desigualdades no acesso aos serviços ^3^ ^,^ ^4^ ^,^ ^5^ ^,^ ^6^ ^,^ ^7^ ^,^ ^8^ ^,^ ^9^ ^,^ ^10^ ^,^ ^11^ ^,^ ^12^ ^,^ ^13^ ^,^ ^14^ ^,^ ^15^ ^,^ ^16^ ^,^ ^17^. Nesse cenário, monitorar tendências de morbimortalidade e assistenciais torna-se fundamental para identificar possíveis mudanças no padrão de utilização dos serviços pós-desastre.

A investigação dos efeitos à saúde em desastres enfrenta limitações, como a ausência de dados primários e a dificuldade de acompanhar longitudinalmente as populações expostas ^18^. Nessas situações, dados secundários dos sistemas nacionais de informação em saúde constituem alternativa relevante, por possibilitarem análises temporais amplas e comparações entre exposições. Contudo, apesar da ampla cobertura territorial, esses sistemas apresentam restrições de qualidade e completude ^5^ ^,^ ^18^ ^,^ ^19^. Em cenários de desastre, são frequentemente a única fonte disponível, dada a interrupção dos serviços e a inviabilidade de inquéritos primários. Estudos prévios após desastres de mineração e eventos naturais no Brasil mostram que esses sistemas conseguem identificar mudanças assistenciais, embora enfrentem desafios como subnotificação e heterogeneidade de registros ^3^ ^,^ ^4^ ^,^ ^8^ ^,^ ^9^ ^,^ ^19^.

Ainda assim, essas bases constituem ferramenta indispensável para a vigilância em saúde e para a produção de evidências em situações de emergência ou desastres de larga escala, sobretudo no Brasil, onde os impactos à saúde tendem a ser subestimados ^5^ ^,^ ^20^.

Diante da magnitude do desastre, do tempo decorrido desde o rompimento da barragem e das potenciais repercussões sobre a assistência em saúde das populações atingidas, torna-se fundamental ampliar o conhecimento sobre o perfil de morbimortalidade e possíveis alterações nos padrões de atendimentos por doenças crônicas e transtornos mentais, portanto, este estudo teve como objetivo analisar o perfil, identificando alterações nos padrões assistenciais de saúde, utilizando-se de dados secundários de acesso público nos municípios atingidos e em municípios utilizados como comparação, com características demográficas semelhantes, contribuindo para o aprimoramento das ações de vigilância pós-desastre.

## Métodos

### Desenho do estudo

É um estudo ecológico analítico, que utilizou dados agregados por município abrangendo o período de 2008 a 2023 para avaliar os impactos na saúde associados ao desastre ambiental decorrente do rompimento da barragem da Samarco. Utilizou-se uma abordagem comparativa entre grupos populacionais expostos e não expostos, com análise temporal segmentada em períodos pré, pós-rompimento e pós-COVID-19.

### População do estudo

**Figura 1:**
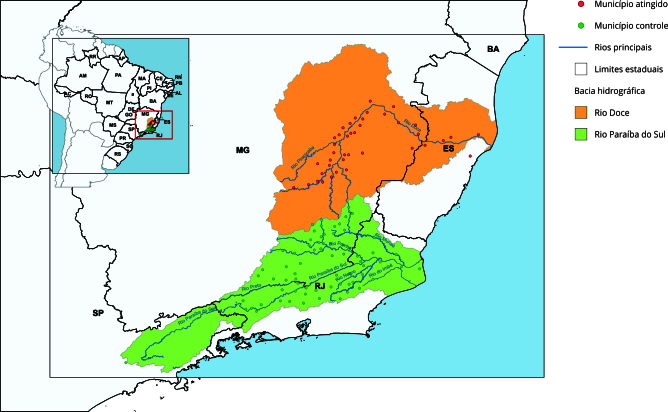
Unidades hidrográficas da Bacia Hidrográfica Atlântico Sudeste, Brasil, e os municípios (atingidos e comparação) da área de estudo.

As bacias do Rio Doce, atingida pelo rompimento da barragem, e do Rio Paraíba do Sul, considerada neste estudo como área de comparação, foram objeto do estudo (Figura 1), sendo duas das principais bacias da macrorregião Sudeste. Fazem parte da região hidrográfica do Atlântico Sudeste, uma das mais importantes do Brasil, abrangendo uma extensa área que se estende por diversas Unidades Federativas, incluindo Espírito Santo, Minas Gerais, Rio de Janeiro e São Paulo, correspondendo a uma área de 229.972km^2^ ^21^.

Fonte: adaptado de Agência Nacional de Águas e Saneamento Básico ^21^ e Comitê para Integração da Bacia Hidrográfica do Rio Paraíba do Sul ^22^.

A escolha da Bacia do Paraíba do Sul para os municípios de comparação baseou-se em critérios ambientais e socioeconômicos, dada a sua vulnerabilidade à degradação ambiental causada pela presença de barragens e usinas hidrelétricas, conforme descrito no Plano Integrado de Recursos Hídricos da bacia (Material Suplementar; https://cadernos.ensp.fiocruz.br/static//arquivo/suppl-e00193725_8182.pdf) ^21^ ^,^ ^22^.

Ao todo, foram considerados na análise do estudo 108 municípios, sendo 40 atingidos pelo rompimento da barragem de Fundão e 68 selecionados como grupo de comparação. Os municípios de comparação foram selecionados devido ao seu reconhecimento como áreas prioritárias para investimento e potencialmente sujeitas às restrições de uso dos recursos hídricos nessas regiões em decorrência de potenciais problemas ambientais identificados, relacionados à concentração de hidrelétricas e de barragem de contenção de rejeitos da mineração ^22^.

### Fontes de dados

Foram utilizados dados secundários agregados provenientes de três sistemas nacionais oficiais de informação em saúde abrangendo o período de 2008 a 2023.

(i) Sistema de Informações Ambulatoriais do Sistema Único de Saúde (SIA/SUS) - base ambulatorial do SUS que reúne dados de todas as produções ambulatoriais realizadas no sistema público de saúde, abrangendo tanto a atenção básica quanto os atendimentos especializados. A coleta de dados é feita por meio dos Boletins de Produção Ambulatorial (BPA), nas versões individual e consolidada. Esses registros contemplam procedimentos realizados em unidades de saúde de diferentes níveis de complexidade, como centros de especialidades, policlínicas, serviços de apoio diagnóstico, unidades ambulatoriais hospitalares e de atenção básica com produção registrada. O SIA/SUS permite a análise detalhada por tipo de procedimento, especialidade, diagnóstico principal (Classificação Internacional de Doenças - 10ª revisão; CID-10), tipo de estabelecimento e município de atendimento ou de residência dos pacientes.

(ii) Sistema de Informações Hospitalares do SUS (SIH/SUS) - base de dados de internações hospitalares: consolidado em 1981, registra as Autorizações de Internação Hospitalar (AIH) realizadas em estabelecimentos de saúde que prestam atendimento pelo SUS. O sistema fornece informações sobre o diagnóstico principal que motivou a internação, o tipo de internação, os procedimentos realizados, o tempo de permanência e o município de residência do paciente.

(iii) Sistema de Informações sobre Mortalidade (SIM) - base de dados de mortalidade: criado em 1975, tem como finalidade a coleta sistemática das informações contidas nas Declarações de Óbito emitidas em todo o território nacional. Permite a análise das causas básicas de morte, local de ocorrência e município de residência do falecido, sendo uma das principais fontes para estudos de mortalidade.

Optamos por analisar os dados tendo como base alguns capítulos e grupos de causas definidos pela CID-10, selecionados por sua relevância na carga de doença no Brasil. Para DCNT, foram considerados os seguintes agravos: doenças do aparelho circulatório (I00-I99), neoplasias (C00-C97), doenças do aparelho respiratório (J30-J98) e diabetes mellitus (E10-E14) ^23^. Para transtornos mentais, foram incluídos os transtornos mentais e comportamentais classificados entre os códigos F00 e F99 da CID-10. Para cada sistema, foram considerados os códigos de variáveis que identificam o diagnóstico principal (CID-10) e o município de residência do paciente ou do falecido.

O detalhamento da amostra final, incluindo os critérios de inclusão e exclusão aplicados a cada base, está apresentado no Material Suplementar (https://cadernos.ensp.fiocruz.br/static//arquivo/suppl-e00193725_8182.pdf) onde são mostradas as etapas de filtragem realizadas nos três sistemas (SIM, SIH/SUS e SIA/SUS).

### Estratificação dos municípios

Os municípios foram estratificados em quintis de população com base nos Censos Demográficos de 2010 e 2022 do Instituto Brasileiro de Geografia e Estatística (IBGE) ^24^, com a finalidade de controlar eventuais disparidades entre os grupos geradas pelos diversos tamanhos populacionais. Utilizaram-se apenas dados censitários, sem projeções intercensitárias. O estrato 1 incluiu municípios com até 5.764 habitantes (9 atingidos e 13 de comparação); o estrato 2, de 5.765 a 10.590 habitantes (10 e 11, respectivamente); o estrato 3, de 10.591 a 17.683 habitantes (5 e 17); o estrato 4, de 17.684 a 41.738 habitantes (8 e 13); e o estrato 5, acima de 41.738 habitantes (8 e 14). Três municípios inicialmente considerados para o grupo de comparação - Juiz de Fora (Minas Gerais), Campos dos Goytacazes (Rio de Janeiro) e Itaquaquecetuba (São Paulo) - foram excluídos por apresentarem população média superior a 300 mil habitantes, o que poderia comprometer a comparabilidade com os municípios atingidos.

Na sequência, os estratos foram qualificados com base em indicadores referentes ao ano de 2014, anterior ao desastre, para evitar influência do evento sobre as características analisadas. Foram utilizados quatro indicadores: população média (2010-2022), como base para a estratificação ^24^; produto interno bruto (PIB) *per capita*, para estimar a condição econômica local ^25^; o Índice FIRJAN de Desenvolvimento Municipal (IFDM), que avalia o desenvolvimento em saúde, educação e renda (quanto maior o valor, melhor o desempenho) ^26^; e as coberturas da Estratégia Saúde da Família (ESF) e da atenção básica (AB), como medidas do alcance da atenção primária no SUS ^27^.

### Análise dos dados

Foi realizada uma análise descritiva da evolução temporal das taxas de atendimentos ambulatoriais, internações hospitalares e óbitos, estratificada pelos desfechos indicados anteriormente e quintil populacional, com o objetivo de comparar os municípios atingidos pelo desastre aos municípios de comparação. A análise considerou três períodos distintos: “antes do rompimento” (2008-2015), “pós-rompimento” (2016-2019) e “pós-COVID-19” (2020-2023). A definição dos períodos foi fundamentada em dois marcos temporais relevantes para o contexto do estudo: o rompimento da barragem de Fundão em 2015, que representa o evento crítico de interesse, e a emergência sanitária da pandemia de COVID-19. Todavia, a escolha desses recortes implica reconhecer a possibilidade de sobreposição de efeitos entre o desastre e a COVID-19, especialmente a partir de 2020, o que limita a atribuição precisa das variações observadas a um único evento e reforça a necessidade de interpretação cautelosa dos resultados. Assim, o período anterior a 2015 foi considerado como base de comparação, ao passo que os dois períodos subsequentes permitiram observar os desdobramentos do desastre e possíveis mudanças associadas à pandemia.

Para qualificar os grupos “atingido” e “comparação” dentro de cada estrato populacional, foram aplicados testes de hipótese conforme a distribuição das variáveis socioeconômicas e de acesso aos serviços de saúde. O objetivo foi descrever possíveis diferenças contextuais relevantes entre os grupos pareados, em linha com abordagens metodológicas utilizadas em estudos de desigualdades sociais em saúde no Brasil e em análises contextuais internacionais ^28^ ^,^ ^29^. O nível de significância adotado foi de p < 0,05. Utilizamos medianas e intervalos interquartis (IQR: 1º ao 3º quartil) para as distribuições assimétricas. Os testes de normalidade foram realizados com o teste de Shapiro-Wilk. As comparações entre municípios atingidos e de comparação foram conduzidas por meio do teste t de Student para distribuições normais e do teste de Mann-Whitney para distribuições assimétricas.

As taxas foram obtidas por meio do quociente entre o número total de eventos registrados (consultas, internações ou óbitos) e a população residente no mesmo grupo e período, multiplicado por 100 mil. Utilizou-se como população de referência a média das populações dos municípios de cada grupo (atingido ou de comparação), calculada com base nos dados dos Censos Demográficos de 2010 e 2022, conforme disponibilizado pelo IBGE ^24^. Em seguida, as taxas calculadas por 100 mil habitantes foram apresentadas por desfechos de saúde, estrato e períodos de análise para estimar os possíveis efeitos da exposição (rompimento da barragem).

Para a comparação entre os grupos, foram calculadas as seguintes medidas:

(i) Risco relativo (RR): razão entre as taxas nos municípios atingidos e nos municípios de comparação. Mede a magnitude da associação entre a exposição e o desfecho de saúde.

RR = (taxa nos atingidos)/(taxa nos municípios de comparação)

(ii) Diferença percentual: expressa a variação proporcional entre as taxas dos grupos, permitindo avaliar tendências de aumento ou redução relativa.

Diferença % atingidos = [(taxa atingidos - taxa comparação)/(taxa comparação)] × 100

Diferença % comparação = [(taxa comparação - taxa atingidos)/(taxa atingidos)] × 100

As análises estatísticas e o processamento dos dados foram realizados por meio dos softwares Microsoft Excel (https://products.office.com/) e R, versão 4.4.0 (http://www.r-project.org). O Excel foi utilizado para a organização dos bancos, cálculo de taxas, agregações e estruturação das tabelas. Já o R foi empregado nas análises estatísticas, incluindo o cálculo de indicadores como RR, diferenças percentuais e respectivos intervalos de 95% de confiança (IC95%), além da elaboração de gráficos e automatização das etapas analíticas. A estimativa dos IC95% permitiu avaliar a precisão das medidas e verificar a existência de sobreposição entre os grupos analisados.

### Aspectos éticos

A análise de dados deste projeto, por utilizar exclusivamente dados secundários agregados, de domínio público e sem identificação de indivíduos, está isenta de apreciação por comitê de ética em pesquisa, não se enquadrando nos termos da *Resolução nº 466/2012* ^30^.

## Resultados

Foram analisados 4.088.875 atendimentos ambulatoriais registrados no SIA/SUS entre 2008 e 2023, após aplicação dos critérios de inclusão. Desses, 2.775.020 (68%) estavam relacionados aos grupos de DCNT contemplados na seleção de diagnósticos e 1.313.855 (32%) a transtornos mentais (Material Suplementar; https://cadernos.ensp.fiocruz.br/static//arquivo/suppl-e00193725_8182.pdf). No SIH/SUS, foram identificadas 1.194.921 internações hospitalares compatíveis, sendo 1.011.292 (85%) por causas associadas às DCNT incluídas e 183.629 (15%) por transtornos mentais. Já no SIM, foram analisados 278.212 óbitos, dos quais 271.874 (98%) correspondiam a causas listadas dentro dos grupos de DCNT selecionados e 6.338 (2%) a transtornos mentais.

A análise da caracterização dos municípios atingidos e de comparação indicou que, para o estrato 1, observou-se diferença estatisticamente significativa no PIB *per capita*, com valores inferiores nos municípios atingidos (R$ 8.350; IQR: 8.091-9.678) em relação aos de comparação (R$ 12.171; IQR: 10.286-14.245; p < 0,01) (Tabela 1). Os demais indicadores - população média, IFDM, cobertura da AB e da ESF - não apresentaram diferenças estatísticas entre os grupos. No estrato 2, os municípios atingidos apresentaram menor população média (6.454; IQR: 5.980-6.977 *vs.* 8.196; IQR: 6.692-8.610; p = 0,02) e PIB *per capita* mais baixo (R$ 8.652; IQR: 7.795-10.425 *vs.* R$ 15.530; IQR: 10.182-18.351; p < 0,01). Os demais indicadores foram semelhantes entre os grupos. No estrato 3, verificou-se diferença significativa no PIB *per capita*, com valores de R$ 10.785 (IQR: 8.030-13.116) entre os atingidos e R$ 15.962 (IQR: 11.719-19.692) entre os de comparação (p = 0,03). População média, IFDM e coberturas de saúde não apresentaram diferenças estatísticas relevantes. No estrato 4, os municípios atingidos apresentaram PIB *per capita* inferior (R$ 12.881; IQR: 10.716-16.129) em relação aos de comparação (R$ 22.405; IQR: 16.499-25.137; p = 0,02). Os demais indicadores não diferiram significativamente. Por fim, no estrato 5, nenhum dos indicadores analisados apresentou diferenças estatisticamente significativas entre os grupos, embora os valores de PIB *per capita* e de população média tenham sido numericamente mais elevados nos municípios atingidos.

**Tabela 1:** Caracterização socioeconômica e de cobertura em saúde dos municípios atingidos e de comparação, estratificados por quintis de população média, Brasil.

Estrato	Indicador	Municípios atingidos n	Municípios de comparação n	Municípios atingidos Média (mínimo-máximo)	Municípios de comparação Média (mínimo-máximo)	Valor de p
1	População	9	13	4.017 (2.910-4.563)	3.324 (2.934-4.491)	0,68
2	População	10	11	6.454 (5.980-6.977)	8.196 (6.692-8.610)	0,02
3	População	5	17	13.495 (11.747-14.950)	11.626 (10.624-13.721)	0,29
4	População	8	13	23.642 (20.494-26.305)	26.718 (21.166-34.660)	0,23
5	População	8	14	102.105 (85.078-173.934)	100.439 (70.479-171.499)	0,78
1	PIB *per capita* (R$)	9	13	8.350 (8.091-9.678)	12.171 (10.286-14.245)	< 0,01
2	PIB *per capita* (R$)	10	11	8.652 (7.795-10.425)	15.530 (10.182-18.351)	< 0,01
3	PIB *per capita* (R$)	5	17	10.785 (8.030-13.116)	15.962 (11.719-19.692)	0,03
4	PIB *per capita* (R$)	8	13	12.881 (10.716-16.129)	22.405 (16.499-25.137)	0,02
5	PIB *per capita* (R$)	8	14	32.281 (22.974-40.217)	22.220 (18.574-29.015)	0,24
1	IFDM (2014)	9	13	0,79 (0,77-0,90)	0,76 (0,74-0,81)	0,29
2	IFDM (2014)	10	11	0,76 (0,72-0,85)	0,71 (0,65-0,74)	0,15
3	IFDM (2014)	5	17	0,79 (0,68-0,90)	0,73 (0,67-0,82)	0,36
4	IFDM (2014)	8	13	0,78 (0,75-0,84)	0,77 (0,73-0,83)	0,69
5	IFDM (2014)	8	14	0,84 (0,82-0,86)	0,82 (0,78-0,89)	0,87
1	Cobertura da AB (%)	9	13	100 (100-100)	100 (100-100)	-
2	Cobertura da AB (%)	10	11	100 (100-100)	100 (100-100)	0,34
3	Cobertura da AB (%)	5	17	100 (100-100)	100 (97-100)	0,57
4	Cobertura da AB (%)	8	13	100 (100-100)	100 (100-100)	0,56
5	Cobertura da AB (%)	8	14	96 (76-100)	90 (62-93)	0,28
1	Cobertura da ESF (%)	9	13	100 (100-100)	100 (100-100)	0,46
2	Cobertura da ESF (%)	10	11	100 (100-100)	100 (100-100)	0,34
3	Cobertura da ESF (%)	5	17	100 (96-100)	100 (97-100)	0,96
4	Cobertura da ESF (%)	8	13	100 (100-100)	100 (83-100)	0,10
5	Cobertura da ESF (%)	8	14	74 (58-93)	74 (52-91)	0,53

AB: atenção básica; ESF: Estratégia Saúde da Família; IFDM: Índice FIRJAN de Desenvolvimento Municipal; PIB: produto interno bruto.

Fonte: Instituto Brasileiro de Geografia e Estatística ^24^ ^,^ ^25^; Sistema FIRJAN ^26^; Ministério da Saúde ^35^.

Em relação aos atendimentos ambulatoriais no SIA/SUS, observou-se um comportamento distinto entre os grupos analisados, com tendência a taxas relativamente mais elevadas entre os municípios atingidos em diversos estratos populacionais no período pós-rompimento, em relação ao período antes do rompimento (Tabela 2). No estrato 1, para diabetes, a diferença relativa foi de 300% nos atingidos (RR = 2,97; IC95%: 1,80-4,90), e os de comparação apresentaram leve redução de 6%. No caso das doenças respiratórias, observou-se aumento de 677% nos atingidos (RR = 1,59; IC95%: 1,30-1,95), frente a 24% nos de comparação. Já as taxas de atendimentos por neoplasias foram 78% superiores nos atingidos (RR = 1,78; IC95%: 1,70-1,87), com aumento de 1.172%, frente a 793% entre os de comparação. Para as doenças circulatórias e transtornos metais, os indicadores não evidenciaram diferenças relevantes entre atingidos e comparação.

**Tabela 2:** Taxas de atendimentos ambulatoriais (por 100 mil habitantes), diferenças percentuais entre municípios atingidos e de comparação, e risco relativo (RR), segundo estrato populacional, grupo de doenças e período.

Estrato	Grupo de doenças	Período *	Taxa		Diferença (%) **		RR (IC95%)
			Municípios atingidos	Municípios de comparação	Municípios atingidos	Municípios de comparação	
1	Circulatório	1	68	394	0	0	0,17 (0,14-0,21)
		2	268	1.230	294	212	0,22 (0,20-0,24)
		3	666	625	879	59	1,07 (1,00-1,14)
2	Circulatório	1	85	290	0	0	0,29 (0,27-0,32)
		2	172	207	102	-29	0,83 (0,76-0,92)
		3	323	557	280	92	0,58 (0,55-0,62)
3	Circulatório	1	48	304	0	0	0,16 (0,14-0,17)
		2	403	488	740	61	0,83 (0,79-0,87)
		3	791	605	1.548	99	1,31 (1,26-1,35) ***
4	Circulatório	1	136	655	0	0	0,21 (0,20-0,21)
		2	408	473	200	-28	0,86 (0,84-0,89)
		3	514	687	278	5	0,75 (0,73-0,77)
5	Circulatório	1	230	612	0	0	0,38 (0,37-0,38)
		2	445	763	93	25	0,58 (0,58-0,59)
		3	780	1.240	239	103	0,63 (0,62-0,64)
1	Diabetes	1	34	49	0	0	0,70 (0,09-5,30)
		2	136	46	300	-6	2,97 (1,80-4,90) ***
		3	158	96	365	96	1,65 (1,23-2,20) ***
2	Diabetes	1	121	20	0	0	6,07 (3,44-10,71) ***
		2	109	22	-10	10	4,91 (2,87-8,40)
		3	100	174	-17	770	0,57 (0,46-0,72)
3	Diabetes	1	17	10	0	0	1,64 (0,74-3,64)
		2	68	40	300	300	1,68 (1,35-2,09) ***
		3	97	32	471	220	2,99 (2,52-3,55) ***
4	Diabetes	1	8	570	0	0	0,01 (0,01-0,02)
		2	49	306	513	-46	0,16 (0,14-0,18)
		3	52	516	550	-9	0,10 (0,09-0,11)
5	Diabetes	1	2	5	0	0	0,41 (0,35-0,49)
		2	39	7	1.850	40	5,60 (5,15-6,08) ***
		3	62	48	3.000	860	1,29 (1,24-1,34) ***
1	Respiratório	1	35	138	0	0	0,26 (0,18-0,37)
		2	272	171	677	24	1,59 (1,30-1,95) ***
		3	245	162	600	17	1,51 (1,29-1,77) ***
2	Respiratório	1	22	238	0	0	0,09 (0,07-0,13)
		2	121	289	450	21	0,42 (0,37-0,49)
		3	114	233	418	-2	0,49 (0,43-0,56)
3	Respiratório	1	52	85	0	0	0,61 (0,54-0,69)
		2	235	73	352	-14	3,19 (2,90-3,51) ***
		3	268	110	415	29	2,44 (2,25-2,64) ***
4	Respiratório	1	36	128	0	0	0,28 (0,26-0,30)
		2	217	76	503	-41	2,85 (2,69-3,02) ***
		3	174	64	383	-50	2,73 (2,57-2,91) ***
5	Respiratório	1	40	199	0	0	0,20 (0,20-0,21)
		2	268	121	570	-39	2,22 (2,18-2,27)
		3	268	199	570	0	1,35 (1,32-1,37) ***
1	Neoplasia	1	134	107	0	0	1,25 (1,10-1,42) ***
		2	1.705	956	1.172	793	1,78 (1,70-1,87) ***
		3	6.729	5.024	4.922	4.595	1,34 (1,31-1,37) ***
2	Neoplasia	1	114	119	0	0	0,96 (0,88-1,04)
		2	1.721	1.046	1.410	779	1,64 (1,59-1,70) ***
		3	3.779	5.291	3.215	4.346	0,71 (0,70-0,73)
3	Neoplasia	1	124	79	0	0	1,58 (1,47-1,69) ***
		2	781	556	530	604	1,41 (1,35-1,46) ***
		3	4.871	2.791	3.828	3.433	1,75 (1,72-1,77) ***
4	Neoplasia	1	139	100	0	0	1,38 (1,33-1,44) ***
		2	814	368	486	268	2,21 (2,15-2,27) ***
		3	3.084	1.278	2.119	1.178	2,41 (2,38-2,45) ***
5	Neoplasia	1	327	1.007	0	0	0,32 (0,32-0,33)
		2	1.876	1.868	474	86	1,00 (1,00-1,01)
		3	3.900	2.847	1.093	183	1,37 (1,36-1,38) ***
1	Mental	1	638	3.592	0	0	0,18 (0,17-0,19)
		2	718	1.165	13	-68	0,62 (0,58-0,65)
		3	616	2.201	-3	-39	0,28 (0,27-0,30)
2	Mental	1	109	527	0	0	0,21 (0,19-0,23)
		2	184	329	69	-38	0,56 (0,52-0,61)
		3	391	8.417	259	1.497	0,05 (0,04-0,05)
3	Mental	1	2.108	990	0	0	2,13 (2,09-2,17) ***
		2	1.837	2.401	-13	143	0,76 (0,75-0,78)
		3	1.081	1.757	-49	77	0,61 (0,60-0,63)
4	Mental	1	653	296	0	0	2,21 (2,16-2,26) ***
		2	1.022	435	57	47	2,35 (2,30-2,41) ***
		3	1.184	685	81	131	1,73 (1,69-1,76) ***
5	Mental	1	687	885	0	0	0,78 (0,77-0,78)
		2	1.708	1.122	149	27	1,52 (1,51-1,53) ***
		3	1.628	1.423	137	61	1,14 (1,14-1,15) ***

Nota: base de dados: Sistema de Informações Ambulatoriais do Sistema Único de Saúde (SIA/SUS).

* Períodos - 1: antes do rompimento, 2: pós-rompimento, 3: pós-COVID-19;

** Diferenças percentuais nas taxas dos períodos (pós-rompimento/pós-COVID-19) em comparação com o período de base (antes do rompimento);

*** Resultados estatisticamente significativos.

No estrato 2, apesar de reduções absolutas nas taxas entre os períodos, observou-se elevada razão de risco para alguns grupos de doenças. Em relação ao grupo diabetes, mesmo com redução percentual entre os períodos, o RR no pós-rompimento ainda foi elevado entre os atingidos (RR = 4,91; IC95%: 2,87-8,40), o que representa uma taxa cerca de 390% superior em relação ao grupo de comparação, considerando o pós-rompimento. Para atendimentos por neoplasias, os municípios atingidos apresentaram taxas 1,64 vez maior no período pós-rompimento em comparação aos municípios de comparação (RR = 1,64; IC95%: 1,59-1,70), com um aumento de 1.410% em relação ao período anterior, frente a uma elevação de 779% nos municípios de comparação. Para os demais grupos de doenças, não foram identificadas diferenças relevantes entre atingidos e comparação.

No estrato 3, os municípios atingidos apresentaram taxas mais elevadas de atendimentos ambulatoriais por diabetes (RR = 1,68; IC95%: 1,35-2,09), doenças respiratórias (RR = 3,19; IC95%: 2,90-3,51) e neoplasias (RR = 1,41; IC95%: 1,35-1,46), com aumentos de 300%, 352% e 530%, respectivamente, no período pós-rompimento em relação ao período anterior. Nos municípios de comparação, os aumentos foram ainda maiores para diabetes (300%) e neoplasias (604%), e as taxas por doenças respiratórias apresentaram redução de 14%. Já nos demais grupos, não foram evidenciadas diferenças relevantes.

No estrato 4, observou-se que as elevações nas taxas entre os atingidos no período pós- rompimento foram de 503% para doenças respiratórias (RR = 2,85; IC95%: 2,69-3,02), 486% para neoplasias (RR = 2,21; IC95%: 2,15-2,27), 57% para transtornos mentais (RR = 2,35; IC95%: 2,30-2,41), em contraste com uma redução de 41% e aumentos de 268% e 47%, respectivamente, nos municípios de comparação em relação ao período anterior, sem evidências relevantes dos demais grupos de doenças.

Já o padrão apresentado no estrato 5 revelou diferenças percentuais igualmente relevantes no período pós-rompimento. Para diabetes, os atingidos apresentaram taxa 1.850% superior (RR = 5,60; IC95%: 5,15-6,08), e os municípios de comparação aumentaram 40%. Para as doenças respiratórias, a diferença percentual foi de 570% entre os atingidos (RR = 2,22; IC95%: 2,18-2,27), frente a uma redução de 39% nos de comparação. No grupo de transtornos mentais, a taxa dos atingidos foi 149% mais alta (RR = 1,52; IC95%: 1,51-1,53), frente a um aumento de 27% entre os não atingidos. Nos demais grupos, não foram observadas diferenças relevantes.

Nas taxas de internações hospitalares registradas no SIH/SUS, embora as variações percentuais tenham sido menos acentuadas, evidenciaram-se diferenças consistentes entre os grupos (Tabela 3). No estrato 3, em relação ao período anterior ao rompimento, as internações por neoplasias foram 60% superiores entre os atingidos e os municípios de comparação apresentaram aumento de 14% (RR = 1,23; IC95%: 1,15-1,31). No estrato 4, as internações por doenças respiratórias apresentaram uma leve redução entre os atingidos (-7%) em relação ao período antes do rompimento, ainda assim com razão de taxas superior (RR = 1,11; IC95%: 1,06-1,17) no pós-rompimento, contrastando com uma redução de 29% nos municípios de comparação. Para neoplasias a taxa de internações foi mais elevada entre os atingidos (47%; RR = 1,34; IC95%: 1,28-1,41), frente a um aumento de 32% nos de comparação. No estrato 5, as internações por neoplasias foram 68% superiores entre os atingidos (RR = 1,25; IC95%: 1,23-1,28), frente a um aumento de 25% no grupo de comparação. Nos demais grupos de doenças não foram observadas diferenças relevantes.

**Tabela 3:** Taxas de internações hospitalares (por 100 mil habitantes), diferenças percentuais entre municípios atingidos e de comparação, e risco relativo (RR), segundo estrato populacional, grupo de doenças e período.

Estrato	Grupo de doenças	Período *	Taxa		Diferença (%) **		RR (IC95%)
			Municípios atingidos	Municípios de comparação	Municípios atingidos	Municípios de comparação	
1	Circulatório	1	349	522	0	0	0,67 (0,63-0,71)
		2	348	548	0	5	0,64 (0,59-0,69)
		3	385	533	10	2	0,72 (0,67-0,78)
2	Circulatório	1	344	668	0	0	0,51 (0,50-0,53)
		2	335	570	-3	-15	0,59 (0,55-0,62)
		3	326	499	-5	-25	0,65 (0,62-0,69)
3	Circulatório	1	380	576	0	0	0,66 (0,64-0,68)
		2	335	569	-12	-1	0,59 (0,56-0,62)
		3	339	530	-11	-8	0,64 (0,61-0,67)
4	Circulatório	1	434	618	0	0	0,70 (0,69-0,72)
		2	508	545	17	-12	0,93 (0,91-0,96)
		3	416	578	-4	-7	0,72 (0,70-0,74)
5	Circulatório	1	327	557	0	0	0,59 (0,58-0,59)
		2	340	494	4	-11	0,69 (0,68-0,70)
		3	348	461	6	-17	0,75 (0,74-0,77)
1	Diabetes	1	63	75	0	0	0,84 (0,71-0,99)
		2	65	74	3	-1	0,87 (0,70-1,09)
		3	47	69	-25	-8	0,69 (0,53-0,89)
2	Diabetes	1	71	103	0	0	0,69 (0,63-0,76)
		2	48	84	-33	-19	0,58 (0,49-0,67)
		3	42	68	-42	-35	0,61 (0,51-0,74)
3	Diabetes	1	42	83	0	0	0,51 (0,46-0,56)
		2	37	78	-13	-6	0,47 (0,40-0,56)
		3	29	71	-31	-15	0,41 (0,35-0,49)
4	Diabetes	1	78	103	0	0	0,76 (0,72-0,79)
		2	77	80	-1	-22	0,97 (0,90-1,04)
		3	70	73	-10	-29	0,96 (0,89-1,03)
5	Diabetes	1	33	48	0	0	0,69 (0,67-0,71)
		2	36	37	8	-22	0,96 (0,92-1,00)
		3	36	41	9	-14	0,87 (0,83-0,91)
1	Respiratório	1	150	152	0	0	0,98 (0,90-1,08)
		2	110	116	-27	-24	0,95 (0,82-1,10)
		3	111	113	-26	-26	0,98 (0,84-1,15)
2	Respiratório	1	139	261	0	0	0,53 (0,50-0,57)
		2	94	202	-33	-23	0,46 (0,42-0,51)
		3	89	133	-36	-49	0,67 (0,60-0,75)
3	Respiratório	1	136	182	0	0	0,74 (0,70-0,79)
		2	124	145	-9	-20	0,85 (0,78-0,93)
		3	97	134	-29	-26	0,72 (0,66-0,79)
4	Respiratório	1	167	198	0	0	0,84 (0,82-0,87)
		2	156	140	-7	-29	1,11 (1,06-1,17) ***
		3	120	148	-28	-25	0,81 (0,77-0,86)
5	Respiratório	1	105	114	0	0	0,92 (0,90-0,94)
		2	85	88	-19	-23	0,96 (0,93-0,99)
		3	93	91	-11	-20	1,02 (1,00-1,05)
1	Neoplasia	1	176	245	0	0	0,72 (0,66-0,78)
		2	251	309	43	26	0,81 (0,74-0,89)
		3	304	306	73	25	0,99 (0,91-1,09)
2	Neoplasia	1	157	225	0	0	0,70 (0,66-0,74)
		2	206	230	31	2	0,90 (0,83-0,97)
		3	238	241	52	7	0,99 (0,92-1,06)
3	Neoplasia	1	145	165	0	0	0,88 (0,83-0,93)
		2	232	189	60	14	1,23 (1,15-1,31) ***
		3	270	206	86	25	1,31 (1,23-1,39) ***
4	Neoplasia	1	133	110	0	0	1,21 (1,16-1,26) ***
		2	195	146	47	32	1,34 (1,28-1,41) ***
		3	214	163	61	48	1,31 (1,26-1,37) ***
5	Neoplasia	1	138	148	0	0	0,93 (0,92-0,95)
		2	231	184	68	25	1,25 (1,23-1,28) ***
		3	250	203	81	37	1,23 (1,21-1,25) ***
1	Mental	1	45	281	0	0	0,16 (0,13-0,19)
		2	42	72	-6	-74	0,59 (0,44-0,78)
		3	44	78	-2	-72	0,57 (0,44-0,74)
2	Mental	1	59	185	0	0	0,32 (0,29-0,35)
		2	58	96	-2	-48	0,60 (0,51-0,71)
		3	50	80	-15	-57	0,62 (0,52-0,74)
3	Mental	1	57	254	0	0	0,22 (0,21-0,24)
		2	43	116	-24	-54	0,37 (0,32-0,43)
		3	56	88	-1	-65	0,63 (0,56-0,72)
4	Mental	1	49	230	0	0	0,21 (0,20-0,23)
		2	77	132	56	-42	0,58 (0,54-0,62)
		3	82	106	66	-54	0,77 (0,72-0,82)
5	Mental	1	60	271	0	0	0,22 (0,22-0,23)
		2	34	185	-44	-32	0,18 (0,18-0,19)
		3	56	148	-6	-45	0,38 (0,37-0,39)

Nota: base de dados: Sistema de Informações Hospitalares do Sistema Único de Saúde (SIH/SUS).

* Períodos - 1: antes do rompimento, 2: pós-rompimento, 3: pós-COVID-19;

** Diferenças percentuais nas taxas dos períodos (pós-rompimento/pós-COVID-19) em comparação com o período de base (antes do rompimento);

*** Resultados estatisticamente significativos.

No SIM, no período pós-rompimento, observaram-se taxas de mortalidade por transtornos mentais mais elevadas entre os municípios atingidos em comparação aos municípios de comparação no mesmo período em relação ao período antes do rompimento (Tabela 4). No estrato 2, o RR foi de 1,57 (IC95%: 1,00-2,45), com aumento de 13% nas taxas entre os atingidos e de 8% entre os municípios de comparação. No estrato 4, o RR foi de 1,44 (IC95%: 1,11-1,86), com aumento de 29% nas taxas entre os atingidos, e os municípios de comparação apresentaram estabilidade. Para os demais grupos de doenças não foram observadas diferenças estatisticamente relevantes.

**Tabela 4:** Taxas de óbitos (por 100 mil habitantes), diferenças percentuais entre municípios atingidos e de comparação, e risco relativo (RR), segundo estrato populacional, grupo de doenças e período.

Estrato	Grupo de doenças	Período *	Taxa		Diferença (%) **		RR (IC95%)
			Municípios atingidos	Municípios de comparação	Municípios atingidos	Municípios de comparação	
1	Circulatório	1	103	125	0	0	0,82 (0,74-0,91)
		2	107	113	4	-10	0,94 (0,81-1,10)
		3	127	137	24	9	0,93 (0,81-1,07)
2	Circulatório	1	106	116	0	0	0,92 (0,85-0,99)
		2	102	119	-5	3	0,86 (0,77-0,95)
		3	113	113	6	-2	1,00 (0,90-1,11)
3	Circulatório	1	84	116	0	0	0,73 (0,68-0,78)
		2	96	119	14	2	0,81 (0,74-0,89)
		3	98	125	16	8	0,78 (0,71-0,86)
4	Circulatório	1	97	133	0	0	0,73 (0,70-0,76)
		2	98	127	1	-4	0,77 (0,73-0,82)
		3	113	133	16	1	0,85 (0,80-0,90)
5	Circulatório	1	78	128	0	0	0,61 (0,60-0,62)
		2	87	133	12	4	0,66 (0,64-0,67)
		3	97	127	24	-1	0,76 (0,74-0,78)
1	Diabetes	1	33	42	0	0	0,78 (0,59-1,03)
		2	31	42	-5	0	0,74 (0,52-1,06)
		3	37	42	12	1	0,86 (0,63-1,18)
2	Diabetes	1	27	26	0	0	1,04 (0,86-1,25)
		2	32	32	19	25	0,99 (0,79-1,25)
		3	29	33	9	30	0,87 (0,69-1,09)
3	Diabetes	1	17	23	0	0	0,75 (0,63-0,89)
		2	18	23	1	-1	0,76 (0,60-0,96)
		3	23	25	34	7	0,94 (0,76-1,15)
4	Diabetes	1	17	23	0	0	0,75 (0,68-0,83)
		2	19	26	12	13	0,74 (0,65-0,85)
		3	24	25	39	11	0,94 (0,83-1,07)
5	Diabetes	1	12	20	0	0	0,58 (0,55-0,61)
		2	15	23	28	13	0,66 (0,62-0,70)
		3	23	24	96	16	0,98 (0,93-1,03)
1	Respiratório	1	38	41	0	0	0,94 (0,74-1,18)
		2	33	43	-13	6	0,77 (0,57-1,05)
		3	44	49	15	19	0,91 (0,69-1,20)
2	Respiratório	1	25	30	0	0	0,84 (0,71-1,00)
		2	29	32	13	7	0,89 (0,71-1,13)
		3	38	27	49	-9	1,41 (1,12-1,72)
3	Respiratório	1	22	28	0	0	0,79 (0,70-0,94)
		2	23	27	5	-4	0,85 (0,72-1,07)
		3	25	28	11	0	0,89 (0,74-1,10)
4	Respiratório	1	21	26	0	0	0,80 (0,75-0,90)
		2	19	26	-9	2	0,74 (0,65-0,84)
		3	25	26	20	2	0,97 (0,86-1,10)
5	Respiratório	1	15	23	0	0	0,64 (0,61-0,67)
		2	16	25	10	6	0,67 (0,63-0,71)
		3	17	24	16	2	0,73 (0,69-0,78)
1	Neoplasia	1	64	68	0	0	0,94 (0,82-1,09)
		2	78	79	22	16	0,99 (0,83-1,19)
		3	81	79	26	17	1,01 (0,85-1,21)
2	Neoplasia	1	57	62	0	0	0,91 (0,82-1,01)
		2	63	64	10	3	0,97 (0,84-1,12)
		3	70	70	24	13	1,00 (0,87-1,15)
3	Neoplasia	1	49	57	0	0	0,86 (0,78-0,95)
		2	67	61	37	8	1,09 (0,97-1,23)
		3	67	70	38	24	0,96 (0,85-1,08)
4	Neoplasia	1	47	56	0	0	0,82 (0,78-0,88)
		2	53	64	13	13	0,83 (0,76-0,90)
		3	60	70	29	25	0,86 (0,79-0,92)
5	Neoplasia	1	47	64	0	0	0,74 (0,72-0,76)
		2	57	74	22	16	0,78 (0,75-0,80)
		3	65	78	38	22	0,84 (0,81-0,86)
1	Mental	1	32	28	0	0	1,14 (0,72-1,81)
		2	36	30	13	7	1,21 (0,69-2,10)
		3	28	29	-12	3	0,97 (0,49-1,95)
2	Mental	1	20	14	0	0	1,50 (1,05-2,15) ***
		2	23	15	13	8	1,57 (1,00-2,45) ***
		3	22	14	8	6	1,52 (0,97-2,39)
3	Mental	1	10	11	0	0	0,86 (0,62-1,20)
		2	11	10	15	-14	1,16 (0,74-1,82)
		3	12	12	27	6	1,04 (0,70-1,56)
4	Mental	1	7	6	0	0	1,11 (0,91-1,36)
		2	9	6	29	0	1,44 (1,11-1,86) ***
		3	10	7	46	4	1,57 (1,23-2,00) ***
5	Mental	1	4	5	0	0	0,75 (0,68-0,82)
		2	4	6	19	25	0,71 (0,63-0,81)
		3	5	7	38	37	0,75 (0,67-0,84)

Nota: base de dados: Sistema de Informações sobre Mortalidade (SIM).

* Períodos - 1: antes do rompimento, 2: pós-rompimento, 3: pós-COVID-19;

** Diferenças percentuais nas taxas dos períodos (pós-rompimento/pós-COVID-19) em comparação com o período de base (antes do rompimento);

*** Resultados estatisticamente significativos.

## Discussão

Evidências científicas demonstram que desastres ambientais desencadeiam uma ampla gama de efeitos adversos à saúde, tanto imediatos quanto persistentes. Entre os mecanismos biológicos estão a exposição a contaminantes ambientais, como metais pesados e poeiras minerais, o estresse crônico prolongado, a desorganização da rede de apoio social e a insegurança alimentar. Esses fatores aumentam a vulnerabilidade a doenças metabólicas, respiratórias, cardiovasculares, câncer e transtornos mentais ^3^ ^,^ ^6^ ^,^ ^7^ ^,^ ^8^ ^,^ ^9^ ^,^ ^10^ ^,^ ^11^ ^,^ ^12^ ^,^ ^13^ ^,^ ^14^ ^,^ ^15^ ^,^ ^16^ ^,^ ^17^ ^,^ ^20^ ^,^ ^31^. Os padrões identificados neste estudo reforçam achados já documentados em outros contextos de emergências ambientais, sugerindo que o desastre de Fundão produziu efeitos duradouros e multifacetados sobre a saúde das populações atingidas ^6^ ^,^ ^12^.

Os resultados referentes à saúde mental são consistentes com a literatura, que identifica os transtornos mentais como uma das principais manifestações de saúde em populações expostas a desastres. Em nosso estudo, no período pós-rompimento, observou-se elevação nos atendimentos ambulatoriais por transtornos mentais nos estratos 4 e 5, com RR de até 2,35 entre os atingidos em comparação ao grupo de comparação (SIA/SUS). No Brasil, após o desastre de Brumadinho, o Projeto Saúde Brumadinho identificou prevalências significativamente elevadas de sofrimento psíquico, depressão, ansiedade e transtorno de estresse pós-traumático (TEPT), reforçando a magnitude dos impactos psicossociais em comunidades atingidas ^8^. De forma semelhante, após o furacão Katrina, nos Estados Unidos, estudos de seguimento demonstraram padrões persistentes de TEPT, transtornos de ansiedade e de humor, além de ideação suicida ^11^. No Japão, após o acidente nuclear de Fukushima, foram relatadas prevalências elevadas de TEPT, depressão e angústia psicológica em diferentes grupos populacionais, incluindo evacuados, mães e trabalhadores da planta nuclear ^32^.

Revisões internacionais, incluindo a experiência de Chernobyl, também confirmam que desastres ambientais e tecnológicos estão associados a aumentos relevantes de transtornos mentais ^12^ e uma metanálise mostrou que cerca de um terço das pessoas expostas a desastres apresentou consequências negativas em saúde mental, sobretudo depressão, ansiedade e TEPT ^11^. No caso de Fundão, avaliações conduzidas pela Fundação Getulio Vargas (FGV) também destacaram os transtornos mentais como dimensão central dos impactos em saúde ^6^ ^,^ ^7^, em consonância com os achados deste estudo. Esses resultados reforçam a necessidade de estratégias territoriais voltadas à saúde mental, incluindo o fortalecimento da Rede de Atenção Psicossocial (RAPS) e o monitoramento em longo prazo dos grupos mais vulneráveis.

Outro achado importante foi a elevação nas taxas de atendimentos por doenças respiratórias em diversos estratos populacionais, como observado nos estratos 1, 3, 4 e 5 para atendimentos ambulatoriais, com risco relativo variando de 1,59 a 3,19. A liberação de milhões de metros cúbicos de rejeitos contendo poeiras minerais e metais pesados, como arsênio, chumbo, manganês e ferro, pode ter desempenhado papel central nesse processo ^13^. Estudos nacionais já haviam indicado associação entre o desastre da barragem de Fundão e o aumento da morbidade respiratória nos municípios atingidos, com destaque para o crescimento de atendimentos ambulatoriais e internações por agravos respiratórios, especialmente em crianças e idosos, conforme apontado pelas análises conduzidas pela FGV ^6^ ^,^ ^7^ e pela Fundação Oswaldo Cruz (Fiocruz) ^9^ no caso de Brumadinho. Além disso, estudo epidemiológico específico identificou maior prevalência de doenças respiratórias crônicas e sintomas respiratórios entre moradores expostos ao desastre de Brumadinho, reforçando a hipótese de que a inalação de material particulado e metais pode agravar quadros respiratórios preexistentes ou induzir novos sintomas ^9^. Experiências internacionais corroboram esses achados: após o atentado ao World Trade Center (Estados Unidos), por exemplo, a exposição a partículas finas esteve diretamente associada ao desenvolvimento de hiper-responsividade das vias aéreas em estudos experimentais ^10^. De forma semelhante, análises ambientais em áreas de mineração no Brasil apontaram risco aumentado de contaminação por metais pesados e seus impactos respiratórios e ambientais ^13^. A ausência de elevação consistente nas taxas de mortalidade observada neste estudo sugere que os efeitos respiratórios se concentraram em quadros de menor gravidade, mas de grande impacto sobre a demanda ambulatorial, em especial na atenção primária.

No grupo diabetes, identificou-se um padrão de elevação nas taxas de atendimentos nos municípios atingidos, sugerindo potenciais impactos sobre a demanda dos serviços de saúde, sobretudo no nível ambulatorial, como evidenciado nos estratos 1, 2, 3 e 5, com RR variando de 1,68 a 5,60. Evidências da literatura indicam que situações de desastre podem agravar condições metabólicas crônicas por meio de múltiplos mecanismos, incluindo estresse psicológico prolongado, insegurança alimentar, mudanças no estilo de vida e interrupções no cuidado continuado ^31^. Após o acidente nuclear de Fukushima (Japão), estudos de coorte evidenciaram aumento significativo da incidência de diabetes e da síndrome metabólica entre os evacuados, acompanhado de elevação de indicadores como índice de massa corporal, circunferência abdominal e glicemia de jejum, em comparação com os não evacuados ^32^. De forma semelhante, após o furacão Katrina pessoas com diabetes enfrentaram dificuldades de acesso a medicamentos, interrupções no fornecimento de insulina e inadequação alimentar em abrigos, resultando em pior controle glicêmico e maior ocorrência de complicações agudas, como hiperglicemia grave e cetoacidose diabética ^33^. No caso de Fundão, esse padrão foi mais evidente nos estratos de menor e maior portes populacionais, em que se observaram aumentos expressivos nas taxas de atendimentos após o rompimento, chegando a 300% no estrato 1 e a 1.850% no estrato 5. Esses achados reforçam que, em contextos de desastre, a combinação entre vulnerabilidade social e fragilidade no acesso contínuo ao cuidado em saúde pode se traduzir em maior procura por consultas, exames e atendimentos de urgência, configurando sobrecarga para os serviços, especialmente os ambulatoriais.

No caso das neoplasias, os aumentos observados nos atendimentos ambulatoriais para os estratos 1, 2, 3 e 4 com variações de RR entre 1,41 e 2,21 devem ser interpretados com cautela, uma vez que se trata de agravos de evolução crônica e de longa latência. Cabe destacar que o SIA/SUS, mesmo sem a utilização das Autorizações de Procedimentos de Alta Complexidade (APAC), abrange não apenas atendimentos na atenção básica, mas também consultas e procedimentos realizados em centros especializados e policlínicas, incluindo seguimento oncológico, rastreamento, diagnóstico, controle de sintomas e cuidados paliativos. Assim, os incrementos identificados não refletem necessariamente a incidência de novos casos de câncer, mas podem indicar maior demanda por serviços especializados, possivelmente associada à reorganização dos fluxos assistenciais após o desastre, ao aumento da procura por diagnóstico em contextos de insegurança e ao acompanhamento de complicações de casos já existentes ^17^. Ainda assim, não se pode desconsiderar a plausibilidade biológica da associação entre a exposição prolongada a metais pesados presentes nos rejeitos da barragem - como arsênio, cádmio e chumbo - e o risco aumentado de alguns tipos de câncer. A literatura aponta que tais contaminantes ambientais podem atuar como agentes carcinogênicos, contribuindo para processos de iniciação e promoção tumoral ^34^. Experiências internacionais em áreas expostas a contaminantes, como Minamata (Japão), Seveso (Itália) e populações impactadas por desastres industriais como Bhopal (Índia), apontam para a necessidade de vigilância contínua, mesmo quando os efeitos só se tornam evidentes após décadas ^15^.

Portanto, os resultados deste estudo devem ser compreendidos menos como reflexo direto da incidência imediata de câncer e mais como um indicador sentinela de maior demanda por diagnóstico, seguimento clínico e cuidado especializado em populações atingidas por desastres ambientais. Esse padrão reforça a importância de estruturar políticas de vigilância oncológica, ampliar o acesso a exames de rastreamento e fortalecer os serviços de referência, de modo a permitir a detecção precoce e o manejo adequado de possíveis efeitos tardios da exposição ambiental.

Embora menos acentuadas do que nos atendimentos ambulatoriais, as internações hospitalares também refletiram diferenças consistentes entre os grupos. No estrato 4, as internações por doenças respiratórias permaneceram mais elevadas entre os atingidos em comparação aos municípios de referência (RR = 1,11; IC95%: 1,06-1,17), mesmo diante de uma redução relativa. Para neoplasias, as internações foram superiores nos estratos 3, 4 e 5, com RR entre 1,23 e 1,34, reforçando a hipótese de maior demanda por serviços hospitalares especializados em áreas afetadas.

No caso da mortalidade, os achados foram pontuais e restritos a determinados estratos, sobretudo em relação aos transtornos mentais nos estratos 2 e 4 com RR de 1,57 e 1,44, respectivamente. Ainda que as taxas sejam baixas, tais diferenças reforçam a necessidade de monitoramento contínuo desses agravos em populações expostas a desastres ambientais.

No conjunto, os resultados deste estudo evidenciam que os municípios atingidos pelo rompimento da barragem de Fundão apresentaram diferenças relevantes nos padrões de morbimortalidade quando comparados aos municípios de perfil semelhante. Embora não seja possível atribuir causalidade direta, os achados sugerem potenciais impactos sobre a demanda dos serviços de saúde, particularmente no nível ambulatorial, em áreas expostas ao desastre. Além disso, diferenças prévias na organização das redes municipais de saúde, como maior disponibilidade na cobertura da atenção primária, na oferta de serviços especializados e na capacidade diagnóstica, nos municípios atingidos, podem ter influenciado a magnitude dessas diferenças, reforçando a necessidade de considerar o contexto assistencial na interpretação dos achados. Esse cenário reforça a importância de compreender os desastres como determinantes sociais da saúde, exigindo políticas públicas que articulem vigilância epidemiológica, fortalecimento da atenção primária, ações intersetoriais e mecanismos de reparação social. A sobreposição com a pandemia de COVID-19 a partir de 2020, embora configure limitação na distinção precisa dos efeitos, também revela a vulnerabilidade acumulada das populações atingidas, que enfrentaram crises sucessivas em curto espaço de tempo ^9^.

Este estudo apresenta limitações que precisam ser consideradas na interpretação dos achados. Em primeiro lugar, trata-se de um delineamento ecológico, que não permite inferências em nível individual. Além disso, os sistemas nacionais de informação em saúde (SIA/SUS, SIH/SUS e SIM) foram concebidos primordialmente para fins administrativos e de financiamento, e não para vigilância epidemiológica em contextos de desastre. Essa característica implica potenciais restrições de qualidade, como subnotificações, inconsistências de preenchimento e variações na cobertura assistencial entre municípios, o que pode afetar a acurácia dos resultados ^18^ ^,^ ^19^. O SIM, por sua vez, embora apresente boa cobertura nacional, depende da qualidade do preenchimento das declarações de óbito, que pode variar regionalmente.

Outra limitação importante refere-se à categorização utilizada. A análise foi realizada por grandes grupos de doenças, conforme a classificação disponível nos sistemas de informação, o que impede avaliações mais refinadas por códigos específicos da CID-10 ou por condições clínicas individualizadas. Além disso, não foi possível incorporar informações sobre determinantes individuais de saúde, como renda, escolaridade, hábitos de vida ou cobertura assistencial real, o que limita a compreensão dos mecanismos que mediam os efeitos observados. De forma adicional, variáveis contextuais relevantes como PIB *per capita*, porte populacional e cobertura da atenção primária não foram incluídas em modelos de ajuste, o que impede controlar potenciais confundimentos estruturais entre os municípios analisados. A ausência de análises multivariadas decorre da natureza descritiva do estudo e da finalidade de monitoramento, mas implica reconhecer que parte das diferenças pode refletir desigualdades prévias na organização das redes de saúde, e não apenas efeitos associados ao desastre. Adicionalmente, potenciais vieses de seleção, decorrentes de diferenças na capacidade de registro entre os municípios e de aferição relacionados à qualidade dos dados, à completude das variáveis e à sensibilidade dos sistemas para captar casos em cenários de desastre, podem ter influenciado as taxas analisadas, reduzindo a validade interna das estimativas. É também necessário reconhecer que parte das diferenças identificadas pode estar associada a mudanças na organização dos serviços de saúde, à sobreposição com a pandemia de COVID-19 ou a outros determinantes socioeconômicos não controlados nesta análise ^18^ ^,^ ^19^.

Essas limitações não invalidam os achados, mas indicam a necessidade de cautela na interpretação e reforçam a importância de estudos futuros com delineamentos longitudinais, metodologias mistas e integração de dados ambientais, clínicos e sociais. Somente abordagens dessa natureza permitirão aprofundar a compreensão dos impactos de longo prazo de desastres ambientais sobre a saúde das populações e apoiar o planejamento de estratégias de reparação, mitigação e vigilância em saúde.

## Conclusão

Os achados deste estudo evidenciam que os desastres ambientais de grande magnitude podem modificar padrões de utilização dos serviços de saúde, gerando pressões adicionais tanto na atenção ambulatorial quanto, em menor escala, no nível hospitalar e na mortalidade por causas específicas. No caso do rompimento da barragem de Fundão, identificaram-se diferenças consistentes entre municípios atingidos e de comparação, sugerindo que a sobrecarga assistencial foi expressa de forma heterogênea entre estratos populacionais e grupos de doenças. É importante destacar que esses resultados derivam de um delineamento ecológico, de modo que as associações observadas refletem padrões populacionais e não permitem inferências sobre riscos individuais ou mecanismos causais diretos.

Esses resultados reforçam a necessidade de fortalecer a resiliência do SUS em regiões expostas a desastres, com ênfase no monitoramento contínuo, na integração das redes assistenciais e na ampliação do acesso a cuidados especializados. É importante destacar que os aumentos observados nas taxas não devem ser interpretados como variações na incidência das doenças, mas como possíveis reflexos de maior demanda assistencial e de reorganizações nos serviços locais após o desastre. A continuidade do cuidado, a atenção psicossocial e o acompanhamento das condições crônicas devem ser priorizados como estratégias centrais para reduzir a sobrecarga e mitigar os impactos prolongados sobre os serviços.

Além disso, o uso de sistemas nacionais de informação em saúde como ferramenta de vigilância em contextos de desastre é promissor, ao permitir a identificação de tendências e a comparação entre diferentes grupos populacionais. Investimentos em análises complementares, com delineamentos mais refinados e integração de dados clínicos, sociais e ambientais, poderão ampliar a compreensão dos efeitos de longo prazo e subsidiar políticas públicas mais equitativas, sustentáveis e intersetoriais de mitigação e reparação. Nesse sentido, embora os resultados reflitam um contexto específico, a identificação de padrões assistenciais e de saúde mental tem potencial para informar estratégias de vigilância ambiental e psicossocial em outros cenários de desastre, oferecendo subsídios para o fortalecimento de políticas de preparação, resposta e monitoramento em territórios vulnerabilizados.

## Data Availability

As fontes de informação utilizadas no estudo estão indicadas no corpo do artigo.

## Material Suplementar

Material Suplementar
