# Supplementary material for: Health surveillance after the Samarco dam disaster: evaluation of standards of care in chronic diseases and mental disorders (2008-2023)
Source: Cad Saude Publica. 2026 Aug 10;42:e00193725. [Article in Portuguese] doi: 10.1590/0102-311XPT193725 (PMC13460898; doi:10.1590/0102-311XPT193725)

MATERIAL SUPLEMENTAR

Quadro S1 Comparação das Semelhanças Ambientais, Demográficas, Econômicas e Sociais entre as Bacias Hidrográficas do Rio Doce e Rio Paraíba do Sul.

**Aspectos**	**Bacia do Rio Doce (Atingidos)**	**Bacia do Rio Paraíba do Sul (Comparação)**
Localização	Região Sudeste do Brasil (MG e ES)	Região Sudeste do Brasil (SP, MG, RJ)
Extensão	Aproximadamente 853 km	Aproximadamente 1.137 km
Clima	Tropical e subtropical úmido	Tropical e subtropical úmido
Bioma	Predominantemente Mata Atlântica e Cerrado	Predominantemente Mata Atlântica e Cerrado
Uso do solo	Agricultura, pecuária, mineração e áreas urbanas	Agricultura, pecuária, indústria e áreas urbanas
População	Cerca de 3,5 milhões	Cerca de 5,5 milhões
IDH (2020)	Médio a alto (0,687 a 0,793)	Médio a alto (0,700 a 0,810)
Atividades econômicas	Agricultura, pecuária, mineração, indústria e serviços	Agricultura, pecuária, indústria, comércio e serviços
Problemas ambientais	Desmatamento, poluição hídrica, assoreamento e erosão	Desmatamento, poluição hídrica, assoreamento e erosão
Gestão de recursos hídricos	Comitês de bacias hidrográficas e Agências Nacional de Águas	Comitês de bacias hidrográficas e Agências Nacional de Águas
Preservação e conservação	Unidades de conservação e projetos de recuperação de áreas degradadas	Unidades de conservação e projetos de recuperação de áreas degradadas
Barragens e hidrelétricas	Barragens de rejeitos (ex: Fundão) e pequenas centrais hidrelétricas (PCH)	Pequenas centrais hidrelétricas (PCH) e barragens de rejeitos

Fonte: Adaptado de Agência Nacional de Águas (ANA) [38] e Comitê para Integração da Bacia Hidrográfica do Rio Paraíba do Sul (CEIVAP) [11].

Quadro S2 Etapas de seleção dos registros de DCNT e Transtornos Mentais nos sistemas SIASUS, SIHSUS e SIM.

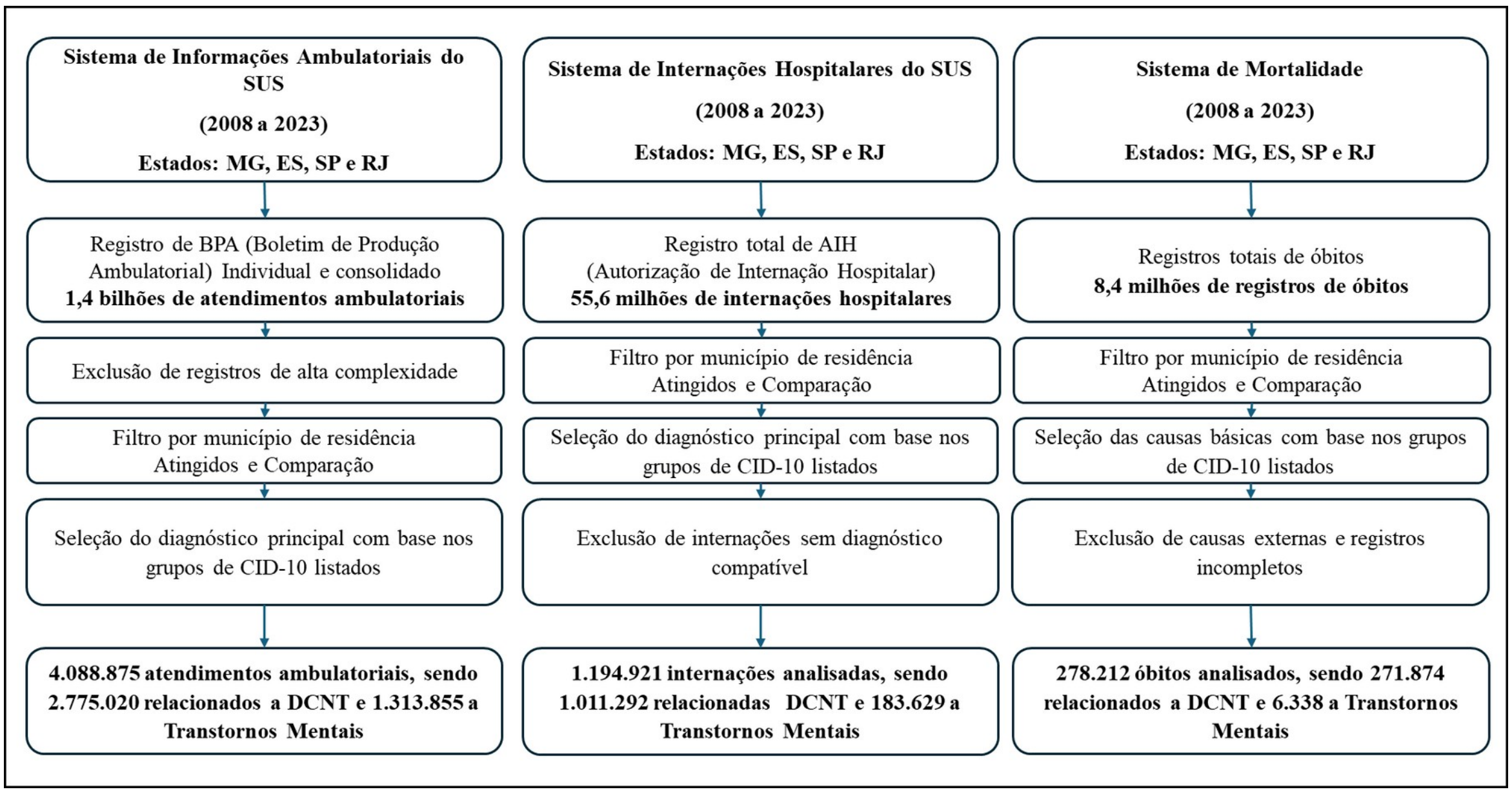

Supplement: Supplementary file 1 [file 1678-4464-csp-42-PT193725-s.pdf]
